# Supplementary material for: PROSER1 mediates TET2 O-GlcNAcylation to regulate DNA demethylation on UTX-dependent enhancers and CpG islands
Source: Life Sci Alliance. 2021 Oct 19;5(1):e202101228. doi: 10.26508/lsa.202101228 (PMC8548262; doi:10.26508/lsa.202101228)
Supplement: Supplementary file 2 [file LSA-2021-01228_TableS2.docx]

Table S2. Primers for molecular cloning.

| Primer Sequence | Primer Name |
| --- | --- |
| ggtaccgagctcggatcctggccaccATGGATTACAAGGATGACGACGATAAGGGCAGCGATTTCCCATCTTGCAGATGTGTAG | TET2 Cys-rich F |
| tgacactatagaatagggccctctagatgcatgctcgagctaAAGCAGCTTAAACTTCCTTGGG | TET2 Cys-rich R |
| ggtaccgagctcggatcctggccaccATGGATTACAAGGATGACGACGATAAGGGCAGCGGGGATGACCCAAAAGAGGAAG | TET2 DSBH1 F |
| tgacactatagaatagggccctctagatgcatgctcgagctaGGAGGAAAGCTTTTCAGCTGC | TET2 DSBH1 R |
| ggtaccgagctcggatcctggccaccATGGATTACAAGGATGACGACGATAAGGGCAGCCTGGAGAACAGCTCAAATAAAAATGAAAAG | TET2 LCI F |
| tgacactatagaatagggccctctagatgcatgctcgagctaGTTGTCCTCTGCACCAGAAGC | TET2 LCI R |
| ggtaccgagctcggatcctggccaccATGGATTACAAGGATGACGACGATAAGGGCAGCGATGAGGTCTGGTCAGACAGC | TET2 DSBH2 F |
| tgacactatagaatagggccctctagatgcatgctcgagctaTATATATCTGTTGTAAGGCCCTGTGACC | TET2 DSBH2 R |
| tgacactatagaatagggccctctagatgcatgctcgagctaGCCATACTTTTCACACTCTTCCTC | TET2 DSBH2-N R |
| ggtaccgagctcggatcctggccaccATGGATTACAAGGATGACGACGATAAGGGCAGCCCAGACTATGTGCCTCAGAAATCC | TET2 DSBH2-C F |
| catggtggccaggatccgagctcgg | pCDNA3 Rev |
| ctcgagcatgcatctagagggcccta | pCDNA3 For |
