# Supplementary material for: PROSER1 mediates TET2 O-GlcNAcylation to regulate DNA demethylation on UTX-dependent enhancers and CpG islands
Source: Life Sci Alliance. 2021 Oct 19;5(1):e202101228. doi: 10.26508/lsa.202101228 (PMC8548262; doi:10.26508/lsa.202101228)
Supplement: Supplementary file 3 [file LSA-2021-01228_TableS3.docx]

Table S3. CRISRPR/Cas9 primers and editing construct sequences.

| Name | Sequence (5’ to 3’) |
| --- | --- |
| hPROSER1 Knockout Reagents | |
| hPROSER1 KO sgRNA spacer  (PC106.PROSER1.g11) | CAAGAAGAUAUCAUAGCAUG |
| PC106.hPROSER1.DS.F2  (NGS overhangs not shown) | AGCTGAGCTCCCAATGTTCA |
| PC106.hPROSER1.DS.R2  (NGS overhangs not shown) | AACCATGAGAAGTTTCATATACAGC |
| hPROSER1 Tagging Reagents | |
| hPROSER1 Tag sgRNA spacer  (CAGE197.PROSER1.g3) | CGUCGGGAUAGUAGUCAAGA |
| CAGE197.5gen.F | CGGCGCAGTGTTGAGTTTAC |
| CAGE197.5junc.R | CCATGTTATCCTCCTCGCCC |
| CAGE197.3junc.F | TACCGATGTGATGGGCATGG |
| CAGE197.3gen.R | TCCGTTTCAGTCAAGGGTCG |
| CAGE197.g3.Flag.HA.NeonGreen.donor  Homology Arms are in lower case | ggcgcgactggctttattgtaggtccccatgagctggtgggggctgtaggggtaaccaggcagggcgtctggcgctgttggggctgcgtttttccggagtccccctatgcgaggccgtgcccctcttcgcccggctcctggtgctccccagccgccggcccgctcccggaagctgcagcagctggtaacaaagagcctgccgggccgcctgctgccggggccgaggtatccaggagtcgggcggagcggctggggatgagcgcgagccggaccatgcgtccgggttcgcggcggtgagggtcgcggccggcggccgaggcgcaggtgcgggtcgagagaccggcgcctccctccccgccgcccgtggactctgagcgcaggtgtttctactctgccggccgctccacctgggaaagcgccttcaagagctacctctggcggcacctggccctcagctgcagggaaaccgcactcaaacctgccgccgagcccgggtttttctgcaaaactcagaggacctggctcttgcccgaggtgccctgaaccattgtgtgaataagctggggaagcctgactggagtgttttttctttttaataaaaagagtttttctgaatctcaatttttagaaattttttttgcgaagtttgcaatactctctacctcttcgcatcctctcttgctcgtggagtctttttcctcctcagctataaatattttttactatcggcggagcagaccagggatgaacgtcttttaattgcaagtataactgttaaaaccacgtcgggatagtagtcaagATGATGGATTACAAGGATGACGACGATAAGGGCAGCTACCCCTACGATGTTCCCGATTACGCTGGCGGCAGCGGCGGCGGCAGCGGCGGCATGGTGAGCAAGGGCGAGGAGGATAACATGGCCTCTCTCCCAGCGACACATGAGTTACACATCTTTGGCTCCATCAACGGTGTGGACTTTGACATGGTGGGTCAGGGCACCGGCAATCCAAATGATGGTTATGAGGAGTTAAACCTGAAGTCCACCAAGGGTGACCTCCAGTTCTCCCCCTGGATTCTGGTCCCTCATATCGGGTATGGCTTCCATCAGTACCTGCCCTACCCTGACGGGATGTCGCCTTTCCAGGCCGCCATGGTAGATGGCTCCGGATACCAAGTCCATCGCACAATGCAGTTTGAAGATGGTGCCTCCCTTACTGTTAACTACCGCTACACCTACGAGGGAAGCCACATCAAAGGAGAGGCCCAGGTGAAGGGGACTGGTTTCCCTGCTGACGGTCCTGTGATGACCAACTCGCTGACCGCTGCGGACTGGTGCAGGTCGAAGAAGACTTACCCCAACGACAAAACCATCATCAGTACCTTTAAGTGGAGTTACACCACTGGAAATGGCAAGCGCTACCGGAGCACTGCGCGGACCACCTACACCTTTGCCAAGCCAATGGCGGCTAACTATCTGAAGAACCAGCCGATGTACGTGTTCCGTAAGACGGAGCTCAAGCACTCCAAGACCGAGCTCAACTTCAAGGAGTGGCAAAAGGCCTTTACCGATGTGATGGGCATGGACGAGCTGTACAAGGGCGGCAGCGGCGGCGGCAGCGGCGGCatggataaaaagtcctttgaaatggtgctggatgaaattagaaaggtaattagaccgattccatgaattattttatgagatgaattctcgtgttttagactctctttgaggtactgtatggagattgtgcagaataagggtcctaggattgccaaattggtttgaattttcacgtgtaatggaggagtttatatcttgatacgtttccagtacaaatagaaattaagttacgttaacgccaaatttgagactcagcacggtgtcgtgggaattacatttgtaaagccctcggttccaaagccggctttacaaatccgtaatagctctgtactttttgggtaaggggtttaatatttttgagtttattgcctcatctttcagtaacacaagagtgactgaaaacgagaggtaatacgcagaaagtttcttttatggtagttattatgatggggttttcaaatctaagcataaggtagagtatatttgttgcgttggccatgcaaaattcgtgtcctgatggtaagtgtggcattaagtttaaagtcacgttcttaggaattggccgtcaataacttgagcttcaaaacttttttctcctgctttccaagtttttgctgctttttgaggctgttgtgtggatcgcatttcccagagtgaaccagacagcaacaattctggaaggtagtcttgagcctaacaatatctcaagcacaaaaataccgagaagttacttattttaaaatgtaaggacttgatgtaccattgttacatcaacacacggtgctttccttcggccttcc |
